# Differential fracture response to traumatic brain injury suggests dominance of neuroinflammatory response in polytrauma

**DOI:** 10.1038/s41598-019-48126-z

**Published:** 2019-08-21

**Authors:** Kazuhito Morioka, Yotvat Marmor, Jeffrey A. Sacramento, Amity Lin, Tiffany Shao, Katherine R. Miclau, Daniel R. Clark, Michael S. Beattie, Ralph S. Marcucio, Theodore Miclau, Adam R. Ferguson, Jacqueline C. Bresnahan, Chelsea S. Bahney

**Affiliations:** 1 Weill Institute for Neurosciences, Brain and Spinal Injury Center (BASIC), Department of Neurological Surgery, University of California, San Francisco (UCSF) & Zuckerberg San Francisco General Hospital (ZSFG), 1001 Potrero Avenue, Building 1, Room 101, San Francisco, CA 94110 USA; 2 Orthopaedic Trauma Institute, Department of Orthopaedic Surgery, University of California, San Francisco (UCSF) & Zuckerberg San Francisco General Hospital (ZSFG), 2550 23rd Street, Building 9, 3rd Floor, San Francisco, CA 94110 USA; 3 San Francisco Veterans Affairs Medical Center, 4150 Clement Street, Building 13, Room 114M, San Francisco, CA 94121 USA; 4 Steadman Philippon Research Institute (SPRI), 181W Meadows Drive, Suite 1000, Vail, CO 81657 USA

**Keywords:** Preclinical research, Experimental models of disease

## Abstract

Polytraumatic injuries, specifically long bone fracture and traumatic brain injury (TBI), frequently occur together. Clinical observation has long held that TBI can accelerate fracture healing, yet the complexity and heterogeneity of these injuries has produced conflicting data with limited information on underlying mechanisms. We developed a murine polytrauma model with TBI and fracture to evaluate healing in a controlled system. Fractures were created both contralateral and ipsilateral to the TBI to test whether differential responses of humoral and/or neuronal systems drove altered healing patterns. Our results show increased bone formation after TBI when injuries occur contralateral to each other, rather than ipsilateral, suggesting a role of the nervous system based on the crossed neuroanatomy of motor and sensory systems. Analysis of the humoral system shows that blood cell counts and inflammatory markers are differentially modulated by polytrauma. A data-driven multivariate analysis integrating all outcome measures showed a distinct pathological state of polytrauma and co-variations between fracture, TBI and systemic markers. Taken together, our results suggest that a contralateral bone fracture and TBI alter the local neuroinflammatory state to accelerate early fracture healing. We believe applying a similar data-driven approach to clinical polytrauma may help to better understand the complicated pathophysiological mechanisms of healing.

## Introduction

In the United States, trauma is the primary cause of fatality in individuals younger than forty-six^1,2^ years old, and in 2006 trauma surpassed cardiovascular disease as the leading medical expenditure^3^. Head injury combined with extremity fracture are a common result of high-energy traumas, such as motor-vehicle accidents, falls, and combat injuries. A recent study found that of 18,404 patients with a femoral shaft fracture, more than one-third sustained a concomitant head or neck injury^4^.

Interestingly, there is a clinical perception that concomitant TBI accelerates bone repair. Clinical studies remain conflicted on the evidence for this phenomenon, partly due to heterogeneous injury patterns and the complex clinical treatment of these polytrauma patients^5^. Moreover, pathophysiological changes that occur as a result of polytrauma are rarely studied and there remains no consensus on how TBI may alter fracture healing at a mechanistic level. The aim of this study is to evaluate mechanisms that may influence healing in a pre-clinical murine model of combined fracture and TBI compared to each injury alone.

When considered alone, mechanical damage from a TBI causes an immediate and direct loss of neural tissue. This damage is followed by a rapid activation of the immune system and a compromised blood-brain-barrier that allows transmigration of leukocytes and activation of microglia^6^. The systemic and local immune activation results in cytokine production that has bimodal impacts on neural tissue: first, by promoting neural damage, approximately days 1–7 in the mouse model, and later repair. Furthermore, the nervous system normally exerts control over the immune system, making the immunological consequences of central nervous system injury complicated^7–9^.

Similarly, fracture healing proceeds through distinct, overlapping processes that require cross-talk across multiple systems^10–13^. Following fracture, a hematoma forms immediately to stop the bleeding, contain debris, and activate a pro-inflammatory response that initiates repair; in the mouse this process occurs from roughly days 3–5 post-fracture^14,15^. Periosteal and endosteal progenitor cells adjacent to the fracture undergo direct differentiation to form bone through intramembranous ossification (days 3–7)^16^. In the fracture gap, periosteal progenitor cells differentiate into chondrocytes and generate a provisional cartilaginous matrix that gives rise to bone indirectly by endochondral ossification (days 5–14)^17^. The cartilage callus matures to bone through transformation of chondrocytes into osteoblasts and matrix remodeling^18–20^. During these remodeling phases, the pro-inflammatory response must resolve or fracture healing will be impaired^15,21^.

A few pre-clinical systems for studying TBI combined with fracture have been published, but similar to clinical trials, there is considerable variation between models and outcomes^5,22,23^. Two recent preclinical studies support evidence for accelerated fracture healing with TBI. These show TBI increased bone volume by μCT at the end stages of fracture repair compared to fracture only, but did not investigate the mechanism(s) driving altered healing^24,25^. Separate studies have shown that serum from patients with TBI can stimulate proliferation of mesenchymal progenitor and/or osteoblast cells lines *in vitro*^26–30^. Candidate screening approaches have identified potential molecular mediators of the osteogenic and/or mitogenic responses, but there is still no consensus that these factors drive the overall response of fracture to TBI *in vivo*^5^.

In this study, our goal was to focus on the earlier stages of fracture repair, days 5–14 post- injury, that are responsible for establishing healing patterns in order to provide more insight to the factors that may drive altered fracture response following combined bone and brain polytrauma. Since it is well-known that the most common deficit following stroke is contralateral hemiparesis, coupled with evidence that changes in the peripheral nervous system can impact fracture healing and heterotopic bone formation^31–33^, we hypothesized that TBI contralateral to a fracture injury would have a differential impact on healing than ipsilateral injuries. If our hypothesis were incorrect, and sidedness does not influence healing outcomes, it would strengthen the argument that systemic factors drive changes in fracture repair following TBI. To test our hypothesis, we created bone and brain injuries either alone, or on the same (ipsilateral) versus opposite (contralateral) sides from each other and compared bone healing, brain lesion size, and inflammatory state. To effectively understand how these complex longitudinal factors work together to drive overall healing response we then integrated all outcome measures into a multidimensional principal component analysis (PCA). This preclinical study is the first to specifically investigate the relative influence of systemic versus neuronal factors in driving bone healing outcomes following TBI and fracture polytrauma and suggests that tracking these factors in a clinical environment are important towards delineating differential healing patterns.

## Results

### Traumatic brain injury accelerates endochondral bone formation during fracture healing without reciprocal effect on brain tissue in contralateral polytrauma

**Figure 1:**
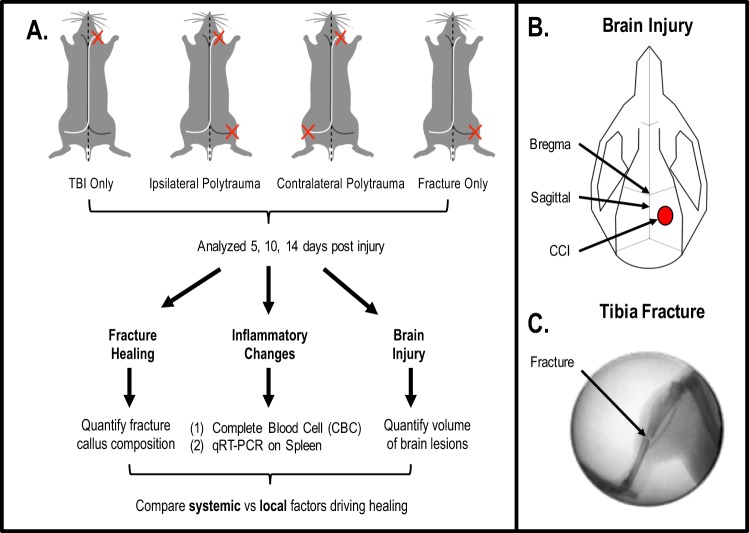
Experimental design overview of traumatic brain injury (TBI) with concomitant tibia fracture. Schematic of experimental design and protocol is described in Materials and Methods. (**A**) The TBI only group included TBI without tibia fracture, the ipsilateral polytrauma group included TBI with ipsilateral tibia fracture, the contralateral polytrauma group included TBI with contralateral tibia fracture, and the fracture only group included tibia fracture without TBI. All assessments were performed at three time points, 5, 10 and 14 days post-injury (n = 5–6/group). (**B**) The diagram of craniotomy site for TBI. (**C**) The representative radiographic image of closed transverse tibia shaft fracture.

To assess both the humoral and neuronal impacts of TBI on fracture healing, we created a novel rodent model which combined a TBI with a concomitant long bone fracture either ipsilateral (same side) or contralateral (opposite side) to the brain lesion (Fig. 1A). Polytrauma injuries were compared to both TBI and fracture only. TBI were produced in the right hemisphere following a craniotomy using an electromagnetic-controlled cortical contusion impactor (CCI)^34–36^ (Fig. 1B). Immediately after the TBI, closed, mid-shaft fractures were created in the left or right tibia using a three-point bending device^17,19^ (Fig. 1C). Animals survived for 5, 10 or 14 days. Blood and spleen were collected to evaluate systemic inflammation. Fracture callus and brain tissue were collected for histological processing and quantification.

**Figure 2:**
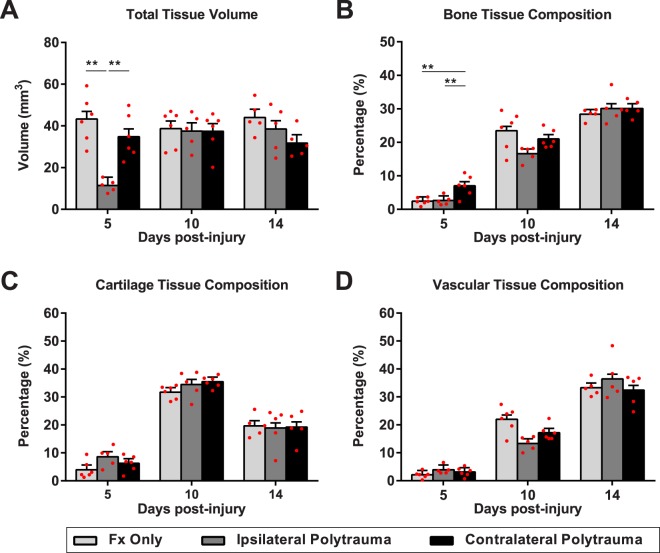
Stereological evaluation of fracture healing. (**A**) Total volume of the fracture callus, (**B**) bone composition in the fracture callus, (**C**) cartilage composition in the fracture callus, (**D**) bone marrow and blood vessel space composition in the fracture callus. Individual data points (red dots) are indicated on the bar graph representing the mean ± standard error of the mean. *p ≤ 0.05, **p ≤ 0.01, ***p ≤ 0.001.

**Figure 3:**
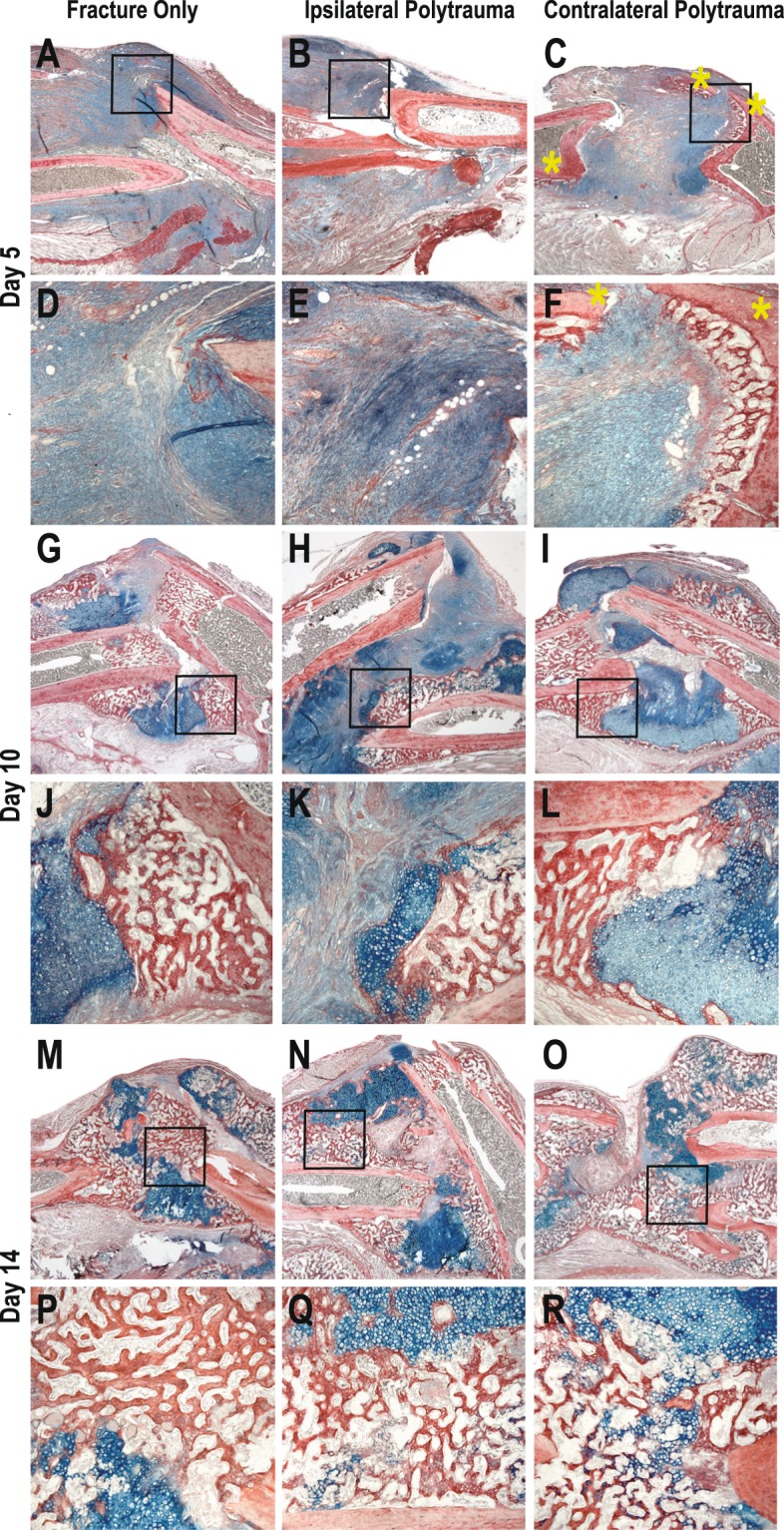
Histology of Bone Fracture. Hall Brundt’s Quadruple Stain (HBQ) histology stains bone red and cartilage blue. (**A**–**F**) 5 Days post-injury, (**G**–**L**) 10 days post-injury, (**M**–**R**) 14 days post-injury.

Fracture callus volume and composition were quantified by blinded reviewers using the Olympus CAST system and Visopharm software to enable an unbiased stereological evaluation of soft and hard tissues within the fracture callus. Fracture alone was compared to the effect of polytrauma. A two-way ANOVA indicates statistically significant differences in fracture healing outcomes considering the main interaction of group crossed with days post-injury for total fracture callus volume (F_(4,39)_ = 6.754, p = 0.0003, η^2^ = 0.292, power = 0.986) and bone composition (F_(4,39)_ = 3.616, p = 0.013, η^2^ = 0.024, power = 0.83). Total callus volume was impacted by both polytrauma and sidedness of the injuries (Fig. 2A and Supplemental Fig. 1). Interestingly, bone composition was not affected by polytrauma overall (F_(1,43)_ = 0.004, p = 0.95, η^2^ = 0, power = 0.05), but only by sidedness of the injury (F_(1,26)_ = 8.297, p = 0.008, η^2^ = 0.242, power = 0.79) (Supplemental Fig. 1). Tukey-Kramer HSD post-hoc testing on the main interaction of group shows that at the earliest time point, 5 days post-injury, there was increased bone formation with polytrauma when the bone injury occurred contralateral to brain injury (Fig. 2B, p = 0.0056). Contralateral polytrauma also had significantly more bone than the ipsilateral polytrauma (p < 0.0105) but did not find a difference between ipsilateral polytrauma and fracture only (p = 0.988). Increased bone composition does not appear to be due to a change in the total fracture callus volume between the contralateral polytrauma group and fracture only (Fig. 2A, p = 0.30), rather increased bone formation was observed along the periosteum adjacent to the fracture gap (Fig. 3C*,F*).

Differential temporal dynamics in total callus volume and bone composition are apparent between fracture and polytrauma groups (Fig. 2). Total callus volume at day 10 post-injury is very similar across all groups. This normalization in volume difference corresponds to the phase in fracture healing where cartilage tissue dominates the fracture callus and we find non-significant differences in cartilage composition with polytrauma (Fig. 2C, F_(4,39)_ = 0.897, p = 0.475, η^2^ = 0.008, power = 0.258). Similarly, post-hoc testing suggests there are no significant difference in bone composition at day 14 (Fig. 2A, F_(2,12)_ = 0.52, η^2^ = 0.246, power = 0.404) Given the significant differences in bone composition at day 5, it suggests that polytrauma is affecting the intramembranous not endochondral healing processes.

**Figure 4:**
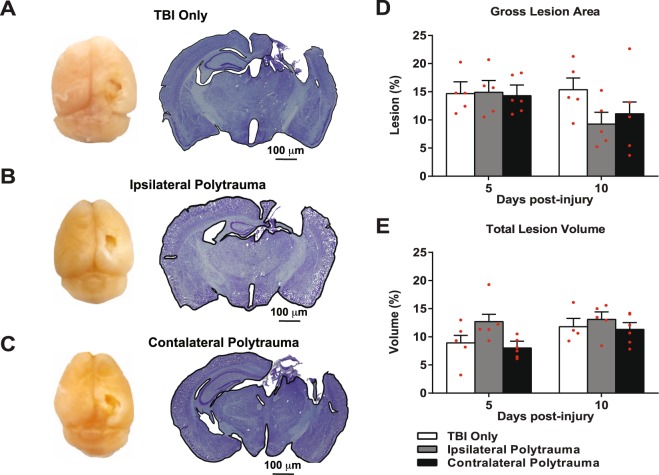
Quantification of Brain Lesions. (**A**–**C**) Representative gross lesions (left panel) and coronal sections through the lesion epicenters (right panel) in the right hemisphere at 10 days after the cortical contusion injury in each group. (**D**,**E**) Gross lesion area (**D**) and total lesion volume (**E**) relative to the whole brain was quantified at 5 and 10 days after injury in each group. Individual data points are indicated by red circles. Two-way ANOVA revealed no statistically significant time × traumatic condition effect among the groups at each time point (p > 0.05). Individual data points are indicated on the bar graph representing the mean ± standard error of the mean.

To evaluate if there was a reciprocal effect of the bone fracture on the brain repair, brain lesion area and volume were quantified using imaging software. Impact data recorded from each animal shows a high level of consistency between all brain injuries (Supplemental Fig. 2). Representative gross images from injured brains 10 days post-injury, show expanded loss of the cortical tissue in the damaged pericontusional region and the absence of swelling (Fig. 4A–C). Coronal cross section at the lesion epicenter in the same mice show diffuse cortical and subcortical atrophy involving the cerebral cortex, corpus callosum, hippocampus, thalamus and hypothalamus in the ipsilateral hemisphere. Based on other studies from our group using similar TBI injury parameters^6,34–36^, differences in gross brain lesion area (F_(2,25)_ = 0.335, p = 0.72, η^2^ = 0.026, power = 0.098) and total brain lesion volume (F_(2,25)_ = 1.344, p = 0.28, η^2^ = 0.097, power = 0.26) between groups and over time were considered practically unimportant and p > 0.05 supports no statistical effect (Fig. 4D,E).

### Differential systemic circulation and inflammatory characteristics in polytrauma

**Figure 5:**
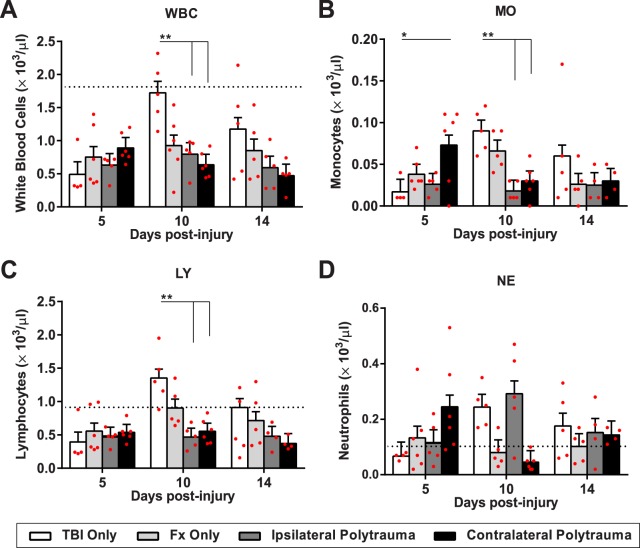
White blood cell differentiation following injury. (**A**) White blood cells (**B**) Monocytes, (**C**) Lymphocytes, (**D**) Neutrophils. Individual data points (red dots) are indicated on the bar graph representing the mean ± standard error of the mean. *p ≤ 0.05, **p ≤ 0.01, ***p ≤ 0.001.

A complete blood count (CBC) was obtained using a multispecies hematology system. Within the broad category of white blood cells (WBC) a two-way ANOVA indicates statistically significant differences with the main interaction of group (WBC; F_(6,51)_ = 3.539, p = 0.005, η^2^ = 0.29, power = 0.92). Individual WBC types, including lymphocytes (LY; F_(6,48)_ = 2.92, p = 0.016, η^2^ = 0.268, power = 0.86), monocytes (MO; F_(6,48)_ = 4.044, p = 0.002, η^2^ = 0.336, power = 0.96), and neutrophils (NE; F_(6,48)_ = 4.485, p = 0.001, η^2^ = 0.36, power = 0.97) were also significantly different (Fig. 5). Within the main effect of group, Tukey-Kramer HSD post-hoc analysis showed that 5 days post-injury, there was an upregulation of monocytes in contralateral polytrauma relative to the TBI only (p = 0.036). At 10 days post-injury, TBI only was significantly different from polytrauma in WBC, lymphocytes and monocytes (p < 0.005). Differences between group at day 14 were much smaller and p > 0.05 supports that early differences in WBC become less important as healing progresses.

WBC differentiation also has a differential temporal dynamic among the injury groups that became evident by plotting these profiles over time (Supplemental Fig. 3). Significant temporal differences are noted in overall WBC (p = 0.02) and LY (p = 0.003) components (Supplemental Fig. 3A–C). Generally, this temporal difference manifests as a unique peak in the TBI only group at 10 days post-injury.

**Figure 6:**
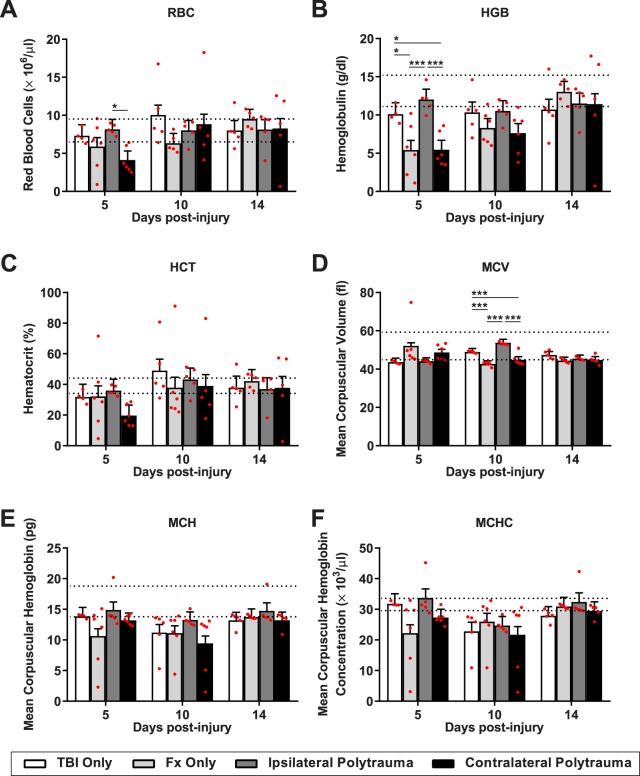
Red blood cell differentiation following injury. (**A**) Red blood cells (RBC). (**B**) Hemoglobulin (HGB). (**C**) Hematocrit (HCT). (**D**) Mean corpus volume (MCV). (**E**) Mean Corpus Hemoglobulin (MCH). (**F**) Mean corpuscular hemoglobin concentration (MCHC); were quantified at 5, 10 and 14 days post-injury in each group. Individual data points (red dots) are indicated on the bar graph representing the mean ± standard error of the mean. *p ≤ 0.05, **p ≤ 0.01, ***p ≤ 0.001.

The red blood cell (RBC) counts indicate blood restoration of regenerative anemia caused by trauma-related hemorrhage. In both the fracture only and contralateral polytrauma groups, average RBC, hemoglobin (HGB), hematocrit (HCT), and mean corpuscular hemoglobin (MCH) levels tended to fall below the normal range at 5 and/or 10 days post-injury according to established clinical pathology reference values (Fig. 6A–F)^37^. Two-way ANOVA finds that the majority of RBC indices do not fall below our significance threshold for post-hoc analysis of p < 0.05, with the exception of hemoglobin (HGB, F_(6,50)_ = 2.27, p = 0.05, η^2^ = 0.211, power = 0.74). By one-way ANOVA, at day 5 post-injury, both RBC (RBC, F_(3,17)_ = 3.85, p = 0.024, η^2^ = 0.122, power = 0.72) and hemoglobin (HGB, F_(3,17)_ = 6.87, p = 0.002, η^2^ = 0.211, power = 0.91) had significant differences by group (Fig. 6A,B).

**Figure 7:**
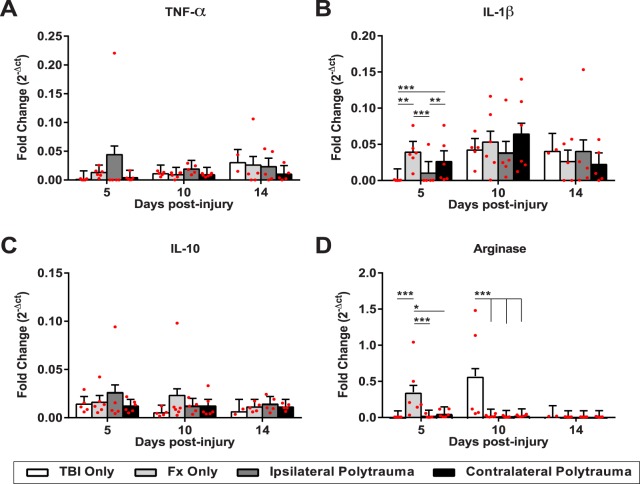
Systemic inflammatory profiles. Gene expression from the spleen tissue for canonical pro-inflammatory markers, (**A**) Tumor necrosis factor-alpha, and (**B**) interleukin-1 beta; and two canonical anti-inflammatory markers (**C**) interleukin-10, and (**D**) arginase. Individual data points (red dots) are indicated on the bar graph representing the mean ± standard error of the mean. *p ≤ 0.05, **p ≤ 0.01, ***p ≤ 0.001.

To understand the systemic inflammatory state, we performed quantitative RT-PCR on spleen tissue. Tumor necrosis factor-α (*Tnfα*) and interleukin-1β (*IL-1β*) are canonical pro-inflammatory markers; arginase (*Arg1*) and interleukin-10 (*IL-10*) are canonical anti-inflammatory markers (Fig. 7). Two-way ANOVA indicated significant changes only in *IL-1β* (F_(11,49)_ = 4.3306, p = 0.0002, η^2^ = 0.257, power = 0.96) and *Arg1* (F_(11,49)_ = 10.8744, p < 0.0001, η^2^ = 0.444, power = 1.0). Tukey-Kramer HSD found fracture only shows higher expression of both *IL-1β* and *Arg1* relative to all other groups 5 days post-injury. By 10 days post-injury the inflammatory response had shifted such that TBI only was the only differential group with higher *Arg1* expression relative to the other groups.

### Multivariate analysis of polytrauma

**Figure 8:**
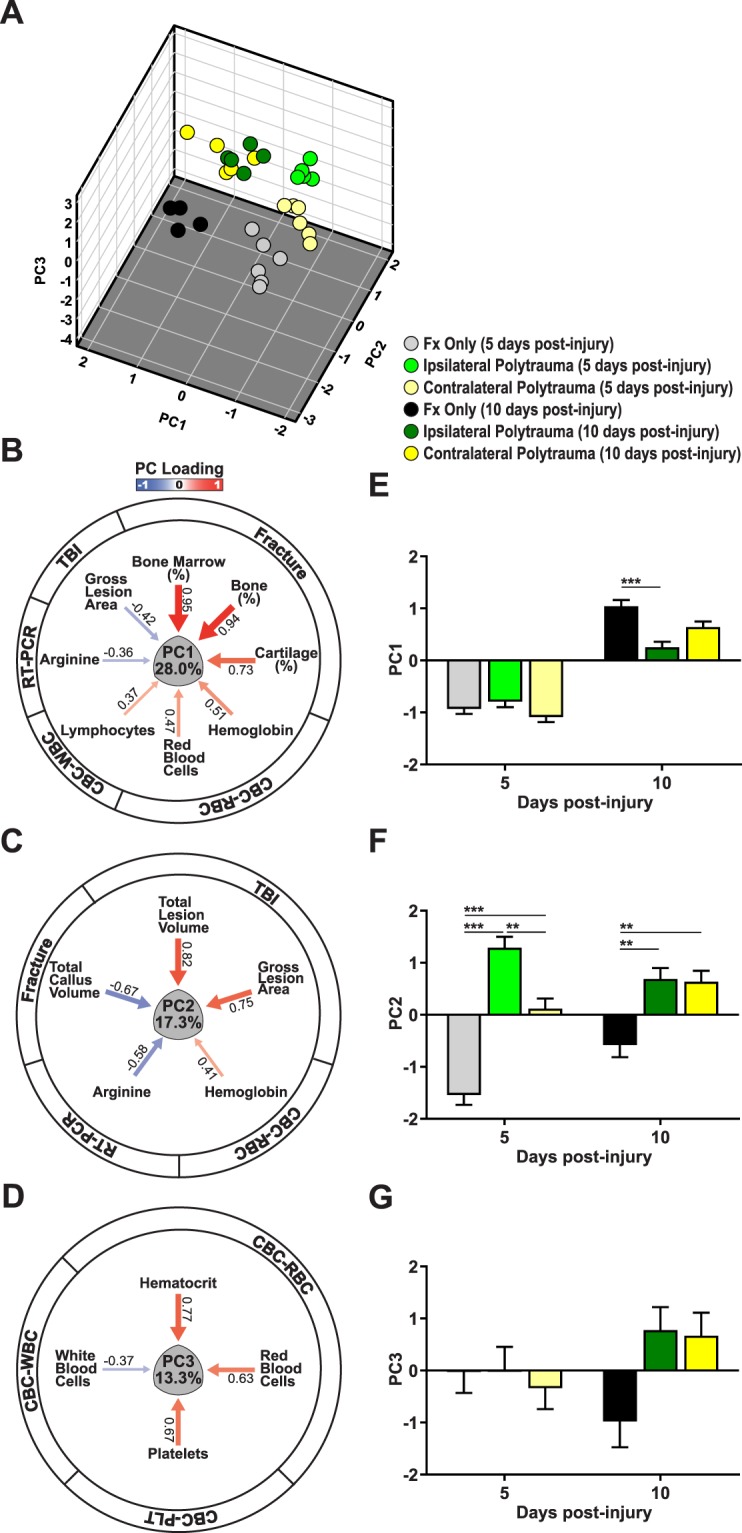
Multivariate principal component analysis (PCA) of polytrauma. The comprehensive correlation among all outcome measures of bone fracture healing, brain injury, systemic circulation and inflammation over time in contralateral and ipsilateral polytrauma was compared to fracture only using PCA. (**A**) The 3-dimensional multivariate syndromic space described by principal components 1–3 (PC1-3). PC scores of individual subjects on PC1-3 extracted from all outcome measures are shown (n = 31; 29 outcome variables). The PC loading pattern resulted in three subject clusters at 5 days post injury revealing a distinct syndromic space for each injury type. At 10 days after injury there are two subject clusters, now distinguishing only between polytrauma and fracture only conditions. (**B**–**D**) PC loading magnitude is indicated by arrow width with heat equivalent to Pearson correlations between the individual variable and the loading value (blue reflects negative and red reflects positive relationships). Exact loading values are shown next to each arrow. PC1 (28% of total variance) reflected the relationship between fracture healing and hematoma formation. PC2 (17.3% of total variance) reflected the relationship between brain injury and blood regeneration. Both PC1 and PC2 outcomes represented the inverse correlation of fracture callus with brain lesion. PC3 (13.3% of total variance) indicated the systemic circulation following injury. (**E–G**) The 2-dimensional PC1-3 loading patterns were analyzed by ANOVA with Tukey’s HSD post-hoc to test for significant differences amongst the main effects at each time point. There is a significant pairwise comparisons between fracture only and ipsilateral polytrauma at 5 days after injury on PC1 (F_(2,25)_ = 9.896, p = 0.001, η^2^  = 0.442, power = 0.971). There are significant pairwise comparisons among each injury condition at 5 days after injury, and significant pairwise comparisons between fracture only and both type of polytrauma at 10 days after injury (F_(2,25)_ = 7.247, p = 0.003, η^2^ = 0.367, power = 0.904). ANOVA on the PCA shows no significant difference between the three injury conditions over time (F_(2,25)_ = 2.891, p = 0.074, η^2^ = 0.188, power = 0.514). *p ≤ 0.05, **p ≤ 0.01, ***p≤.

A principal component analysis (PCA) was completed to provide an unbiased comprehensive assessment of variation given the complexity of our experimental model and the cross-system outcomes. Specifically, PCA enabled us to reduce a large set of variables to a smaller set most likely to contain the meaningful parameters and understand which outcome measures have meaningful co-variation. This was specifically done to complement the discrete hypothesis testing facilitated by classic ANOVA and avoid simplistic dichotomization as statistically significant or not given the limitations of our sample size. The three fracture groups were eligible for the data-driven multivariate analysis since they included all outcome measurements. The PCA revealed three principal components (PCs) that together accounted for 58.6% of the total variance (Supplementary Fig. 4). PC scores of individual subjects on PC1-3 were plotted within the 3-dimentional syndromic space (Fig. 8A).

PC1 accounted for 28% of the total variation (Supplementary Fig. 4A,B) and largely explained variation in fracture healing (Fig. 8B and Supplementary Fig. 4C). Loading values for PC1 were strongly positive for the fracture callus measurements (bone volume, bone composition, marrow composition, cartilage composition) and these responses co-varied with measurements of the hematoma formation (LY, RBC, HGB, HCT). Fracture callus responses negatively correlated with arginase and brain gross lesion area, indicating that accelerated fracture healing was associated with a smaller TBI and less of an anti-inflammatory response. We then assigned individual subject PC scores on PC1 and used ANOVA and Tukeys HSD post-hoc testing to assess the effect of injury condition on PC1 as an ensemble outcome. Statistical analysis of PC1 suggest that fracture healing response in ipsilateral polytrauma is different from contralateral polytrauma (Fig. 8E, F_(2,25)_ = 9.896, p = 0.001, η^2^  = 0.442, power = 0.971).

PC2 accounted for 17.3% of the total variance and the largest positive loading values were associated with the TBI measurements (CCI depth and velocity, gross lesion area, total lesion volume), showing co-variation with blood regeneration (Hb, MCH, mean corpuscular hemoglobin concentration; MCHC), and negative correlation with total fracture callus volume and anti-inflammatory response in the same manner as PC1 (Fig. 8C and Supplementary Fig. 3C). As confirmation that PC2 largely describes the brain injury measurements, loading values for fracture only form a distinct group in PC2 (Fig. 8F, F_(2,25)_ = 7.247, p = 0.003, η^2^  = 0.367, power = 0.904). Further, we again find a differential response between ipsilateral and contralateral polytrauma (Fig. 8E,F). PC3 explains 13.3% of total variance and is associated with systemic inflammation and circulation, including, the hematoma formation and blood regeneration related to injury (Fig. 8D and Supplementary Fig. 3C). Statistical analysis showed no significant pairwise comparisons among the three fracture groups over time (Fig. 8G, F_(2,25)_ = 2.891, p = 0.074, η^2^  = 0.188, power = 0.514).

## Discussion

Irrespective of the tissue damaged, traumatic injuries typically follow a similar healing cascade that begins with mechanical damage to the tissue and activation of a pro-inflammatory response that enables a subsequent reparative phase. Generally speaking, the level and extent of repair are a function of the presence of local tissue resident progenitor cells that can orchestrate tissue regeneration and the ability resolve the pro-inflammatory response. Tissues known to have a strong regenerative capacity capable of restoring form and function to injured tissue include, bone, skin, muscle, liver, and intestine, among others. However, tissues such as brain, cartilage, ligaments/tendons, and lung, have little to no innate regenerative capacity and either fail to close a defect or repair with a fibrotic scar that lacks native function.

Within individual tissue types preclinical animal models have enabled significant progress in understanding the cellular and molecular mechanisms that regulate the reparative process. In murine fracture healing, the pro-inflammatory response occurs during the first week of healing and our previous work has demonstrated that macrophages are critical both in the activation and resolution of inflammation that regulates bone healing^15,21,38,39^. This pro-inflammatory state sets up osteogenic differentiation and intramembranous repair along the periosteum between days 3–7 post-injury. Chondrogenic differentiation for endochondral repair within the fracture gap occurrs primarily between days 5–14, roughly correlating with the resolution of the pro-inflammatory state through preferential activation of an anti-inflammatory macrophage state (“M2”)^11,15,17,40^. In the murine TBI model, controlled cortical injuries causes an immediate loss of neural tissue, rapid activation of the immune system and a temporary breakdown in the blood-brain-barrier to produces a peripheral response through the transmigration of pro-inflammatory leukocytes and microglia within the first week post-injury^6,35^. Interestingly, within the first week following TBI there is a simultaneous production of pro- (“M1”) and anti- (“M2”) inflammatory molecular mediators creating a highly complex inflammatory milieu and suggesting that microglia/macrophages display a mix phenotype and do not discretely polarize to a “M1-only” or “M2-only” state^41,42^ Consequently, the timepoints chosen in this study were designed to capture the early pro-inflammatory phase (day 5) of both injuries and subsequent key regenerative phases in fracture healing (days 10 and 14).

Treatment of polytraumatized patients is challenging because injuries affecting multiple organ systems results in higher mortality and poorer prognosis^22,43^. Further, sustaining multiple traumatic injuries could produce reciprocal impacts on healing progression compared to either injury alone. Understanding the basis for changes in healing patterns following polytrauma therefore becomes complicated by the requirement for cross-disciplinary expertise. TBI and long bone fracture are two organ systems often injured in conjunction. Clinical observations suggest that there is an acceleration in fracture healing with TBI, but strong evidence for this correlation is still lacking.

In the past three decades, a total of 41 clinical and preclinical studies, as well as number comprehensive review articles^5,22,23^, have been published assessing the correlation between TBI and accelerated fracture healing. As of 2015, approximately half of published studies found a significant association between concomitant TBI and enhanced fracture repair, whereas the data from the other half of the studies are inconclusive^5^. To better control for clinical variation, recent preclinical studies have emerged with evidence for enhanced fracture repair demonstrated by earlier bridging callus formation, a two-fold shorter time to union, and increased mean callus thickness^24,25,44^. In addition to faster callus formation, fractures with TBI also present with a higher bone mineral density^25,44^ and increased torsional strength^25^, suggesting more robust repair. Despite a significant body of research, clear evidence and mechanistic data supporting this phenomenon remain unclear. It is also interesting to note that traumatic brain injuries have been shown to have the opposite effect on bone homeostasis, inducing osteoporosis and osteopenia^29,45–50^.

With this study, our goal was to establish a robust and comprehensive data set in the mouse model that would allow us to mechanistically probe the major factors underlying TBI-associated accelerated fracture repair with the intent of discovering potentially novel opportunities for therapeutic intervention of poor fracture healing. Existing research studies have typically focused on a single aspect of TBI-fracture polytrauma, such as hormones, growth factors, metabolism, or inflammatory cells. Cumulatively, these studies have found correlations between healing and specific candidate molecules, such as, leptin^51–53^, prolactin^54^, stem cell derived factor-1^55^, or basic fibroblast growth factor^56^ that may contribute to accelerated fracture repair. Others suggest altered cytokine expression may alter the inflammatory state^57,58^. And a number of *in vitro* studies have demonstrated that serum from TBI-fracture patients accelerates cell proliferation relative to fracture-only serum^26–30^.

Since a pro-inflammatory response is one of the initial steps in healing, we investigated both systemic circulation and inflammatory condition post-injury. Previous research on murine fractures has suggested that hematoma formation and a robust pro-inflammatory response from 3–5 days post-injury is necessary to initiate bone healing, while 10–14 days post-injury is when the callus transitions from cartilage to bone and, at this time, inflammation needs to resolve for normal healing^14,59,60^. In this study, we found that the inflammatory state of mice with polytrauma was differentially modulated relative to either TBI or fracture only. Not only were the CBC profiles different from each other at a specific time point, but they also showed differential temporal dynamics. Using spleen tissue to access specific markers of pro- (*TNFα*, *IL-1β*) versus anti- (*IL-10*, *Arg*) inflammatory markers, we found the fracture only group showed up-regulation of both *IL-1β* and *Arg* relative to other groups immediately after injury, while the TBI only group had the strongest anti-inflammatory (*Arg*) response. Overall these data align with recent reports suggesting concurrent expression responses within both pro- and anti-inflammatory arms and illustrate that the classic *in vitro* nomenclature to delineate macrophage polarity is overly simplistic and that *in vivo* mixed phenotypes are common due to the complex signaling events surrounding them^42^. These complex inflammatory responses emphasize the need to employ comprehensive approaches such as multivariate PCA which revealed the strongly positive relationship between measurements associated with hematoma formation (lymphocytes, red blood cells, hemoglobin, hematocrit) and fracture healing (PC1). We also found the increased bone formation with polytrauma only at the early time point, again supporting the importance of this initial healing response.

The other key factor we found influencing fracture response to TBI was the orientation of the injuries. If humoral factors drove the fracture healing response to TBI, then having the injuries ipsilateral versus contralateral would not matter. However, we found accelerated bone healing occurred most significantly with contralateral injuries. This suggests that some neuronal mechanism may be causing contralateral activation of fracture healing, possibly due to the anatomical crossing of the fibers of the corticospinal tracts from one side of the central nervous system to the other near the junction of the medulla and the spinal cord (“decussation of the pyramids”). Our data also showed that polytrauma more significantly influenced bone composition compared to cartilage composition. A lack of innervation in cartilage would therefore support a mechanistic role of the nerve in intramembranous rather than endochondral repair. The clinical impact of sidedness in bone-brain polytrauma has not previously been considered and recording/tracking this data may provide new insight into the complex and heterogenous responses currently observed. Previous studies have also postulated that TBI induces changes in the central and peripheral nervous system in a manner that positively impacted fracture healing^32,33,44,61^ and homeostatsis^49,50,62^.

One possible mechanism by which the peripheral nerve may influence fracture healing directly is through an interaction with the periosteum. Nerves run throughout the bone and there is a nerve situated directly under the periosteum. Osteochondral progenitor cells in the periosteum are established as the direct source of the cells that generate the fracture callus^16,63^, and our histomorphometry indicates increased periosteal bone formation 5 days post-injury. It is possible that the nerve activates this more robust bone formation either through stimulating proliferation of the periosteal progenitor cells or increasing the concentration of a local osteogenic factor^26,27,29^. A few studies have specifically identified that TBI produces changes in the neuropeptide calcitonin gene-related protein, which can act as a potent vasodilator^33,44^. While we did not see any overall indication of changes in vascularity associated with polytrauma, previous work has shown that vascular endothelial cells are a major source of osteogenic factors that can stimulate fracture healing and damage to the periosteal blood supply causes significant apoptosis in the periosteum which contributes to delayed healing^18,19,64^.

Alternative explanations revolve around the potential for TBI to generate a local change to the neuro-inflammatory state. In this study, our data suggest that formation of a larger hematoma with a more robust early activation of the pro-inflammatory cascade in contralateral polytrauma is positively correlated with better fracture healing, validating published work demonstrating that removal of the hematoma impairs fracture healing^60^. Interestingly, another group has proposed that the underlying mechanism for ectopic bone formation following TBI is that the blood-brain-barrier breakdown causes peripheral nerves to release osteoprogenitor cells from peripheral nerves to establish new bone formation^31,32,65^.

The extent of brain damage also may be important to the fracture healing response. In our experimental design induction of the TBI was controlled using an electromagnetic device with a programmed impact; however, actual measurements of the severity of the CCI and size of the brain lesion were negatively associated with bone formation in our PCA. Increased impact velocity and depth, corresponded to increased brain lesion, and these factors were negatively correlated with fracture healing, indicating that accelerated fracture healing is more likely with smaller brain injuries. Taken together this suggests that severity of the brain injury influences the degree of change in bone formation and helps to explain why there is a broad spectrum of phenotypes reported in the clinical literature.

In conclusion, our preclinical study provides increased evidence that TBI can stimulate bone formation during fracture healing. Importantly, our *in vivo* study was designed to test, for the first time, if laterality of the polytrauma influenced healing outcomes. We show that brain and bone injuries contralateral to each other result in increased bone formation suggesting that neuronal alterations from this injury pattern may mechanistically initiate repair. Furthermore, by taking a robust and unbiased statistical approach to analyzing our data we were able to look at co-variation through a principal component analysis and found that increased pro-inflammatory responses were also positively correlated with bone formation. Taken together, our experimental model design and approach to global data analysis supports that neuroinflammatory changes may establish the accelerated fracture response to TBI and generates a platform for investigation into the specific molecular factors underlying these changes. The long-term goal of this mechanistic work is to translate the basic science of naturally occurring accelerated fracture healing, such as seen here with brain-bone polytrauma, into novel therapeutic approaches to treat the estimated 3 million fractures per year that exhibit recalcitrant healing.

## Materials and Methods

### Traumatic brain injuries (TBI)

All murine procedures were approved by the UCSF Institutional Animal Care and Use Committee (IACUC) and performed in compliance with NIH guidelines. Adult male C57BL/6J mice (10–14 weeks old, Jackson #000664) were randomly divided four groups: (1) TBI without fracture, “TBI Only”; (2) TBI with ipsilateral fracture, “Ipsilateral Polytrauma”; (3) TBI with contralateral fracture, “Contralateral Polytrauma”; and (4) Fracture without TBI, “Fx Only”. TBI were created using an electronic cortical contusion impactor device (“CCI”; model 6.3, Custom Design & Fabrication Inc.) as described previously^34,35^. Briefly, the skull was secured in a stereotaxic frame (David Kopf Instruments) under isoflurane anesthesia. Craniotomies were centered over the right hemisphere with a calibrated manipulator arm 2.5 mm caudal to Bregma and 3.0 mm right of the sagittal suture, then a 5 mm diameter trephine defect was created with a high-speed rotary hand-piece (MH-170, Foredom Electric Co., Bethel, CT; Fig. 1B). The CCI was fitted with a 3.0 mm rounded metal impactor tip, angled at 21-degrees. Impactor was zeroed the brain surface with impact parameters of 2.0 mm deep, 150 ms dwell time, and 4 m/s velocity. Following injury, the wound was irrigated with saline and bone chips removed, then a Gelfoam^®^ sponge (Pfizer) was placed over the bone defect and the skin was sutured. Mice received a peri-operative dose of sustained-release buprenorphine HCl (1.2 mg/kg) as an analgesic and cefazolin (50 mg/kg) as an antibiotic. Of note is that sustained-release buprenorphine HCl was specifically chosen for this study due to clinical and preclinical data suggesting non-steroidal anti-inflammatory drugs (NSAIDs) delay fracture healing^66^; however, long acting buprenorphine in the presence of traumatic injury has been reported to alter cardiovascular responses and subsequent inflammation^67^. Per IACUC stipulations analgesics are required for all preclinical studies, but by keeping the analgesic treatment consistent across groups we aimed to discern differential responses between treatment groups.

### Fractures (Fx)

Immediately after brain injury, a mid-shaft tibia fracture was created either ipsilateral (right side) or contralateral (left side) to TBI. To simulate a traumatic fracture, a custom apparatus that drops a 460 g weight from a height of 14 cm onto the impactor head, generated a closed, transverse fracture as described (Fig. 1C)^17,19,21^. Fractures were confirmed by manual palpitation and fluoroscopy (Hologic Fluoroscan Premier C-Arm Imaging System, Model #QES115-036: 48 kV and 0.021 mA). Post-operatively mice were returned to their home cages on a heating pad and recovery closely monitored. Animals were socially housed and allowed to ambulate freely until experimental end-points 5, 10, or 14 days post-injury.

### Complete blood count (CBC)

At euthanasia, the mice were given an intraperitoneal injection of 250–400 mg/kg tribromoethanol as terminal anesthesia, and when the animal was areflexive, an incision was made to expose the heart. Blood was drawn from the left ventricle via cardiac puncture with a 20-gauge needle. The blood was maintained on ice with ethylenediamine tetraacetic acid (K_2_EDTA) and transported to the UCSF Mouse Pathology Core facility for a complete blood count (CBC) within 24 hours using Multispecies Hematology System (HV950FS, Drew Scientific).

### Systemic inflammation

Spleen tissue was collected into RNA*later*^*®*^ (#R0901, Sigma-Aldrich), stored in −20 °C until transferred to TRIzol^TM^ Reagent (#15596026, Invitrogen^TM^, Thermo Fisher Scientific) for mRNA isolation in accordance with manufacturer’s protocol. cDNA was reverse transcribed with the iSscript^TM^ cDNA Synthesis Kit (#1708890, Bio-Rad Laboratories) and treated with DNA-*free*^TM^ (#AM1906, Invitrogen^TM^, Thermo Fisher Scientific). Quantitative RT-PCR was performed using SYBR^®^ Green Primers (Table 1) and RT^2^ SYBR^®^ Green qPCR Mastermix (#330509, QIAGEN GmbH, Hilden, Germany) on a C1000 Touch^TM^ Thermal Cycler (Bio-Rad Laboratories.) run to 40 cycles. Relative gene expression was calculated by normalizing to the housekeeping gene GAPDH (ΔC_T_). Fold change was calculated as 2^−ΔCT^.

**Table 1:** Primer Sequences.

Housekeeping	mGAPDH Forward	5′-AGCCTCGTCCCGTAGACAAAAT-3′
	mGAPDH Reverse	5′-CCGTGAGTGGAGTCATACTGGA-3′
Pro-Inflammatory	TNFα Forward	5′-TGCCTATGTCTCAGCCTCTTC-3′
	TNFα Reverse	5′-GAGGCCATTTGGGAACTTCT-3′
	IL-1β Forward	5′-TGTAATGAAAGACGGCACACC-3′
	IL-1β Reverse	5′-TCTTCTTTGGGTATTGCTTGG-3′
Anti-Inflammatory	ARG1 Forward	5′-GAACACGGCAGTGGCTTTAAC-3′
	ARG1 Reverse	5′-TGCTTAGCTCTGTCTGCTTTGC-3′
	IL-10 Forward	5′-GCCAAGCCTTATCGGAAATG-3′
	IL-10 Reverse	5′-CACCCAGGGAATTCAAATGC-3′

### Quantifying area and volume of brain lesion

Blood and spleen were collected from each animal before perfusion with 0.1 M phosphate-buffered saline (PBS) and 4% paraformaldehyde (PFA; pH 7.4) at 11.5 ml/min for 15 minutes. Brains and tibias were collected immediately after perfusion for histological analyses. Brains were post-fixed at 4 °C overnight and went through 3 changes of 30% sucrose in PBS over 5 days. Each brain was photographed from a top view from a fixed distance (9.15 cm) with a ruler in each frame. Brain gross lesion area was identified by tracing both the edge of the lesion area and the edge of the whole brain area from the brain top view images using Adobe Photoshop CS5. The brain gross lesion area, expressed in mm^2^, was calculated by dividing the number of pixels in the gross lesion area by the number of pixels in 1 square mm as referenced by the ruler in each image. The relative brain gross lesion area to the whole brain was calculated as the number of pixels in the gross lesion area divided by the number of pixels of the whole brain^68^.

**Figure 9:**
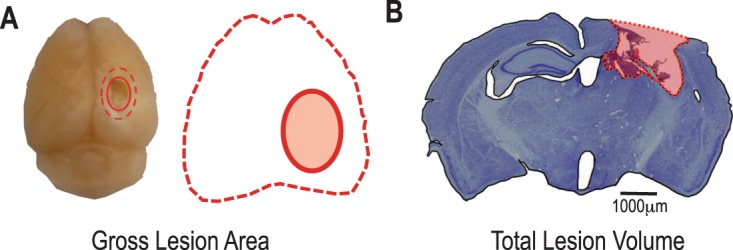
Schematic representation of (**A**) gross lesion area and (**B**) total lesion volume measurements made in the brain following controlled cortical contusion.

Each brain was embedded in Tissue-Tek® O.C.T. compound (Sakura Finetek) and quick frozen in a dry ice chamber. Tissue was cut in 30 μm serial sections on a Shandon Cryotome FSE (Thermo Fisher Scientific) and left to dry overnight before storage at −80 °C. Brain sections were stained with thionin and the extent of the brain injury was quantified using histopathology to determine lesion volume. The lesion epicenter was identified as the section with the greatest amount of tissue loss. Bright-field images, beginning with every 3rd section away (720 μm) from the lesion epicenter until no injured tissue was present, were acquired in 2× magnification and analyzed by the imaging software program Analyzer^TM^ (BZ-9000 Generation II). Lesion and intact brain parenchyma were differentiated and a dividing line through the central sulcus produced ipsilateral and contralateral areas. Spared brain volume, expressed in mm^3^, was calculated by taking the summation of each spared area multiplied by the distance between sections^53^. The total lesion volume was determined by subtracting the ipsilateral cortical volume from the contralateral cortical volume^69^. The relative total lesion volume to the whole brain volume was calculated by dividing the total lesion volume by the total brain volume (Fig. 9). Evaluation was performed blind to experimental conditions.

### Quantifying fracture healing

Fractured tibiae were collected free of skin, washed in PBS, then fixed in 4% PFA (pH 7.2–7.4) overnight, before decalcifying in 19% EDTA (pH 7.4) at 4 °C for 14–18 days. Samples were then dehydrated through a graded ethanol series and embedded in paraffin for histology. Tibiae were serially sectioned (10 μm) through the entire fracture callus using a microtome (Leica Microsystems), three sections placed per slide, and tissue was stained with Hall’s Brundt’s Quadruple (HBQ) stain to demark cartilage in blue and bone in red.

To determine the volume and the composition of the fracture callus, bone, cartilage, vascular tissue, and fibrous tissue were quantified at each time point using 40× magnification and stereology system (Olympus BX51 microscope and CAST stereology system) with the image analysis software (Visiopharm Integrator System version 6.10) as described previously^25,54^. Briefly, bright-field images, beginning with every 10th section away (300 μm) from edge to edge of the fracture callus, were acquired at 2× magnification, and the region of interest (ROI) was defined.by the entire outlines. Tissue composition within the ROI was quantified using automated uniform random sampling to meet or exceeds the established principles for deriving accurate and precise estimates using stereology^70^. Cell identity within each random sampling domain was determined at 20× magnification according to standard histological staining patterns and morphology^25^. Vascular tissue was considered as any portion of the fracture callus that fell within a blood vessel or marrow space of new bone. Finally, all composition measurement was calculated as each fracture tissue volume divided by total callus volume. Evaluation was performed by reviewers blinded to experimental conditions.

### Statistical analyses

In an effort to be fully transparent and accelerate progress our data is available for data sharing on our website: https://scicrunch.org/odc-tbi. All bar and line graphs were made using GraphPad Prism version 8.01 software for Mac (GraphPad Software) to include individual data points (red circles) along with group bars representing the estimated marginal means and the standard error in general linear models (GLM), which were calculated by IBM SPSS Statistics for Windows, version 25.0 (IBM Corp., Armonk, NY, USA). Two-way analysis of variance (ANOVA) was used for multiple longitudinal comparisons using JMP^®^ 13 (SAS Institute) with the main effects of group (polytrauma combined, ipsilateral polytrauma, contralateral polytrauma, fracture only, TBI only) and time considered. F-ratios are reported with numerator and denominator degrees of freedom in subscript for all effects meeting the type I error rate of p < 0.05. One-way ANOVA with post-hoc analysis was used for multiple conditional comparisons followed by Tukey-Kramer Honestly Significant Difference (HSD) test. Principal component analysis (PCA) was performed using eigenvalue decomposition of the cross-correlation matrix of all outcome measures over time in SPSS for syndromic analysis of neurotrauma as described previously^41,71–73^. The outcome measurements from fracture callus in the TBI only group were input as a missing value rather than zero, and the brain lesion in the fracture only group were input as zero due to the intact brain for the validity of statistical analysis. Listwise deletion was used for missing value in the PCA. The syndromic outcome space was plotted using PC1-3 axes without the factor rotation using GPL code written within IBM SPSS v.25 syntax. Each PC reflects an orthogonal linear combination of the variables that accounts for the maximum amount of the total variance in all outcome measures. Number of principal components (PCs) were determined according to the criteria: (1) the Kaiser rule, retaining PCs with eigenvalues greater than 1; (2) the Cattell rule, retaining principal components above the elbow in the scree plot; (3) PC over-determination, retaining components with at least four PC loading values above |0.6|. PC scores were calculated using the regression method. All PC loading values above |0.3| were retained for PC interpretation. The validity of the PC loading pattern was assessed using GLM on the PCA derived scores for ANOVA followed by Tukey’s HSD post-hoc test. Effect size is reported as eta-squared and the precise observed power is reported for ANOVAs performed on PC scores. In all graphs, a statistically significant relationship among the groups for all outcome measures was indicated with a bar and an asterisk according to the following probabilities: *p ≤ 0.05, **p ≤ 0.01, ***p ≤ 0.001.

## Supplementary information

Supplementary Figures
