# Supplementary material for: Differential fracture response to traumatic brain injury suggests dominance of neuroinflammatory response in polytrauma
Source: Sci Rep. 2019 Aug 21;9:12199. doi: 10.1038/s41598-019-48126-z (PMC6704103; doi:10.1038/s41598-019-48126-z)
Supplement: Supplementary file 1 — Supplementary Figures [file 41598_2019_48126_MOESM1_ESM.pdf]

Differential fracture response to traumatic brain injury suggests dominance of neuroinflammatory response in polytrauma

Keywords
Fracture, Traumatic Brain Injury, Polytrauma

Kazuhito Morioka[1,2], Yotvat Marmor[2], Jeffrey A. Sacramento[1], Amity Lin[1], Tiffany Shao[2], Katherine R. Miclau[2], Dan Clark[2], Michael S. Beattie[1], Ralph S Marcucio[2], Theodore Miclau III[2], Adam R. Ferguson[1,3], Jacqueline C. Bresnahan[1*], Chelsea S. Bahney[2,4*]

Affiliations
[1]Weill Institute for Neurosciences, Brain and Spinal Injury Center (BASIC), Department of Neurological Surgery, University of California, San Francisco (UCSF) & Zuckerberg San Francisco General Hospital (ZSFG)
1001 Potrero Avenue, Building 1, Room 101, San Francisco, CA 94110, USA

[2]Orthopaedic Trauma Institute, Department of Orthopaedic Surgery, University of California, San Francisco (UCSF) & Zuckerberg San Francisco General Hospital (ZSFG), CA, United States
2550 23rd Street, Building 9, 3rd Floor, San Francisco, CA 94110, USA

[3]San Francisco Veterans Affairs Medical Center, CA, United States
4150 Clement Street, Building 13, Room 114M, San Francisco, CA 94121, USA

[4]Steadman Philippon Research Institute (SPRI)
181 W Meadows Drive, Suite 1000
Vail, CO 81657 USA

Contributions
K.M., M.S.B, A.R.F., R.S.M., T.M., J.C.B. and C.S.B. designed the experiments; K.M., Y.M., J.A.S., A.L., T.S., D.C, and C.S.B. executed the experiments; K.M., Y.M., J.A.S., A.L., T.S., K.R.M., D.C. and C.S.B. performed analysis of data; K.M., C.S.B and A.R.F. curated the data, and designed and implemented the analysis; M.S.B., A.R.F., R.S.M., T.M., J.C.B., and C.S.B. provided financial support and materials for the studies; K.M., A.R.F., J.C.B. and C.S.B. wrote and edited the manuscript, with comments from all authors.

Corresponding authors
Correspondence to Chelsea S. Bahney (cbahney@sprivail.org) and Jacqueline C. Bresnahan (Jacqueline.Bresnahan@ucsf.edu).

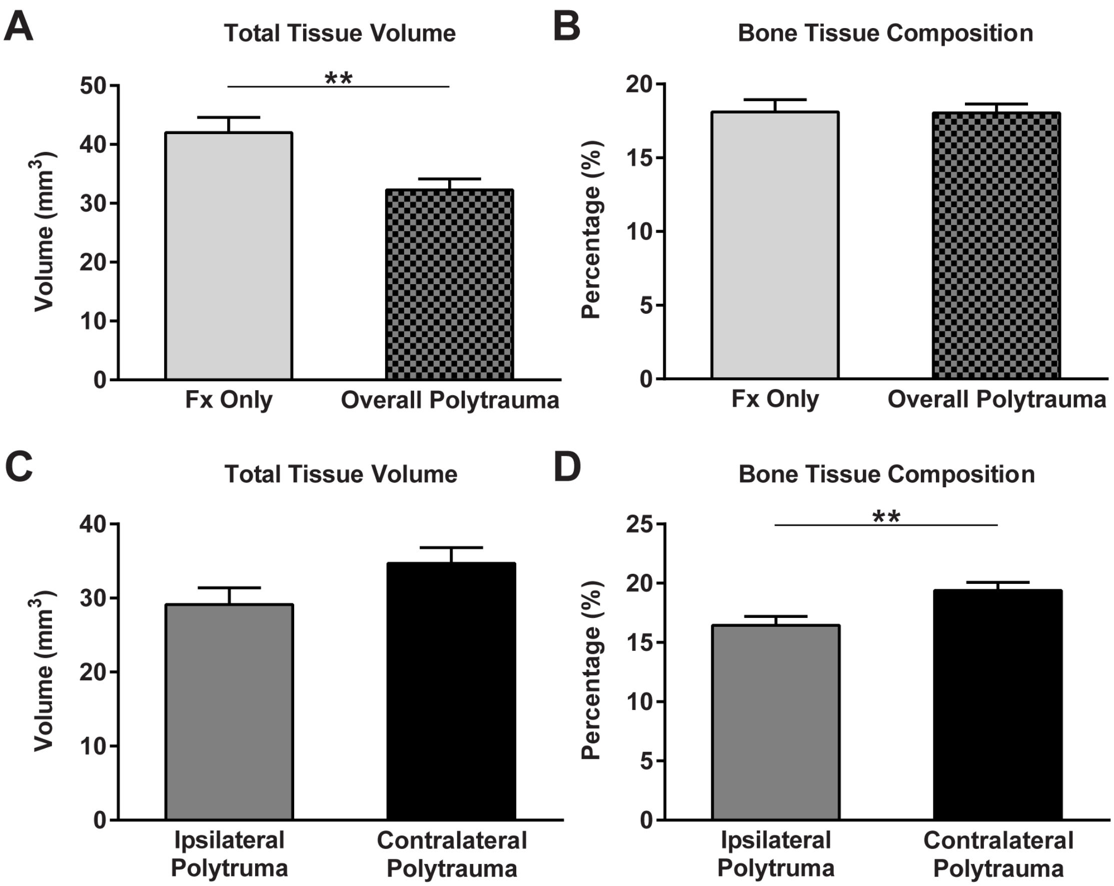

Supplementary Figure 1. Synthetic effects of traumatic brain injury on fracture healing. (A) Total volume of the fracture callus and **(B)** bone composition in the fracture callus between the fracture only group (n=17) and the overall polytrauma group (total n=32; the ipsilateral polytrauma group, n=15, plus the contralateral polytrauma group, n=17) during days 5-14 post-injury. The main effect of two-way ANOVA revealed statistically significant effect of traumatic brain injury on total volume of the fracture callus ($F_{(1, 43)}$ = 9.283, p = 0.004, , η^2 = 0.178, power = 0.846). **(C)** Total volume of the fracture callus and **(D)** bone composition in the fracture callus between the ipsilateral polytrauma group (n=15) and the contralateral polytrauma group (n=17) during days 5-14 post-injury. The main effect of two-way ANOVA revealed statistically significant lateral effect of traumatic brain injury on bone composition in the fracture callus ($F_{(1, 26)}$ = 8.297, p = 0.008, η^2 = 0.242, power = 0.792). The bar graph represents the mean ± standard error of the mean. * = p ≤ 0.05, ** = p ≤ 0.01, *** = p ≤ 0.001.

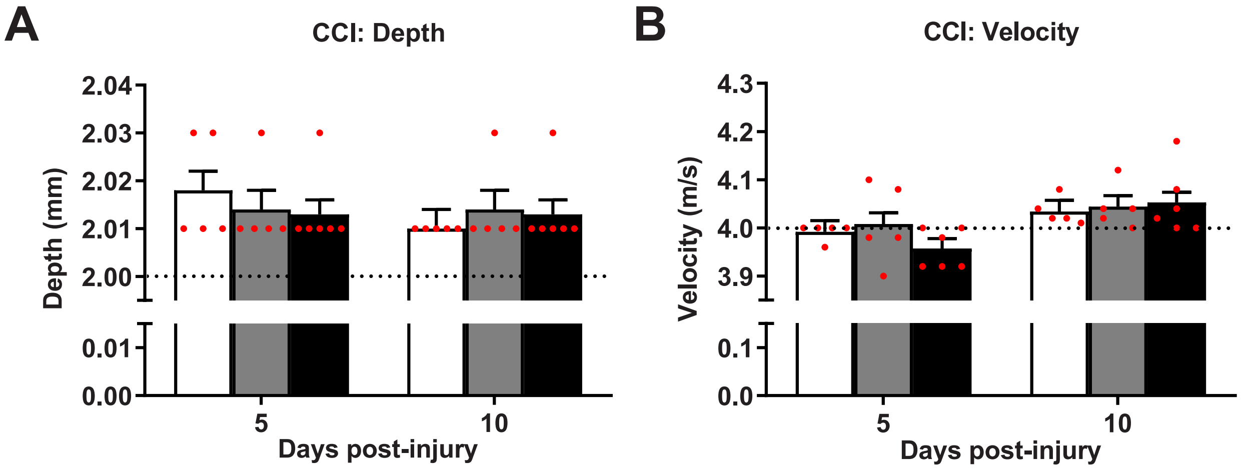

Supplementary Figure 2. The biomechanical conditions of traumatic brain injury. The cortical contusion injury was generated using a controlled cortical contusion impactor (CCI) with a depth setting of 2.0 mm, a dwell time of 150 ms, and a velocity of 4 m/s. These input settings are represented by a horizontal dotted line. Actual **(A)** depth and **(B)** velocity recordings from each subject support that the animals received a similar initial impact ($P > 0.05$).

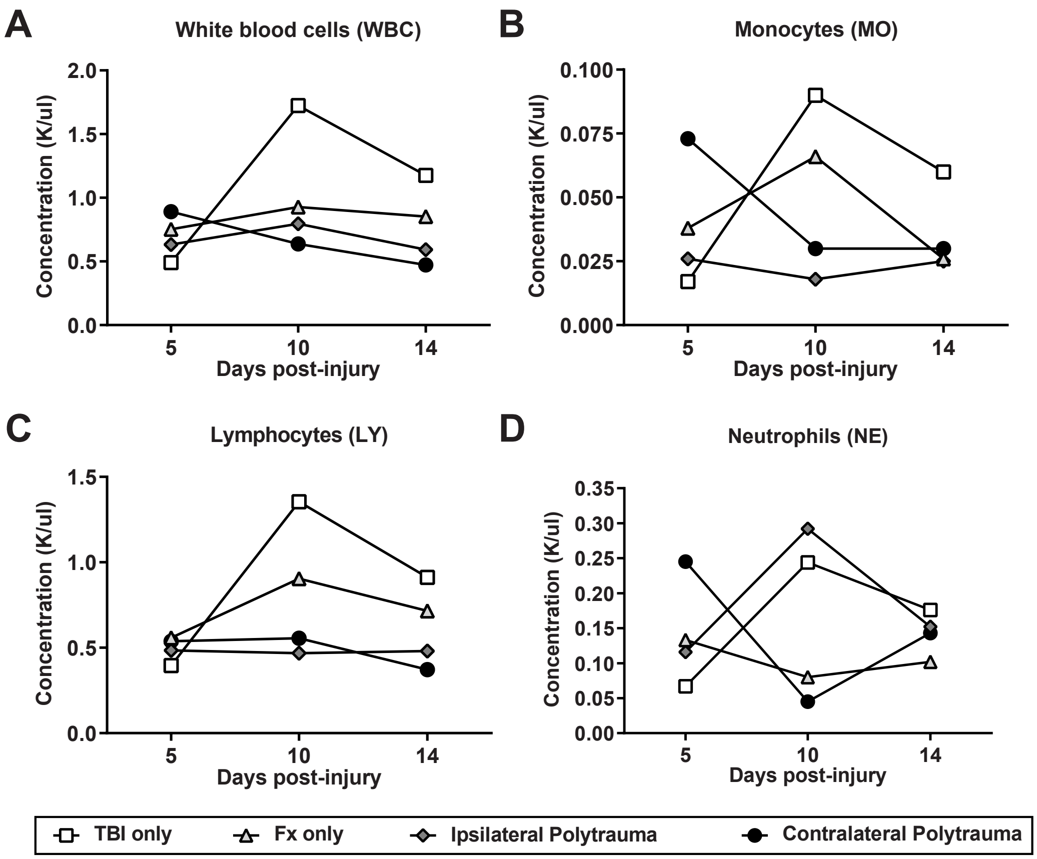

Supplemental Figure 3. White blood cell differentiation over time. **(A)** White blood cells **(B)** Neutrophils, **(C)** Lymphocytes, **(D)** Monocytes was quantified at 5, 10 and 14 days post-injury in each group.

A

Principal Component	Eigenvalues			Extraction Sums of Squared Loadings		
	Total	% of Variance	Cumulative %	Total	% of Variance	Cumulative %
1	8.12	28.00	28.00	8.12	28.00	28.00
2	5.03	17.33	45.33	5.03	17.33	45.33
3	3.84	13.25	58.58	3.84	13.25	58.58
4	2.57	8.87	67.46	2.57	8.87	67.46
5	2.45	8.46	75.92	2.45	8.46	75.92
6	1.63	5.63	81.55	1.63	5.63	81.55
7	1.42	4.88	86.43	1.42	4.88	86.43
8	0.88	3.04	89.47			
9	0.69	2.36	91.83			
10	0.57	1.95	93.78			

B

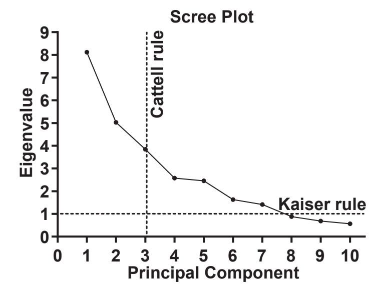

C

Outcome Variables			PC1	PC2	PC3
Fracture	Tissue Volume	Total Callus Volume (mm^3)	0.39	-0.67	-0.05
		Bone Volume (mm^3)	0.95	-0.06	-0.06
		Cartilage Volume (mm^3)	0.79	0.11	0.10
		Bone Marrow/Vascular Tissue Volume (mm^3)	0.95	-0.12	-0.07
		Fibrous Volume (mm^3)	-0.65	-0.63	-0.05
	Tissue Composition	Bone Composition (%)	0.94	0.06	-0.02
		Cartilage Composition (%)	0.73	0.30	0.18
		Bone Marrow/Vascular tissue Composition (%)	0.95	-0.02	-0.04
		Fibrous Composition (%)	-0.95	-0.14	-0.06
Complete Blood Count	White Blood Cell Count	White Blood Cells (WBC) (K/ul)	0.17	-0.21	-0.37
		Neutrophils (NE) (K/ul)	-0.24	0.10	-0.03
		Lymphocytes (LY) (K/ul)	0.37	-0.26	-0.38
		Monocytes (MO) (K/ul)	-0.22	-0.21	-0.43
		Eosinophils (EO) (K/ul)	-0.46	-0.36	-0.10
	Red Blood Cell Indices	Red Blood Cells (RBC) (M/ul)	0.47	0.01	0.63
		Hemoglobin (HGB) (g/dl)	0.51	0.41	-0.01
		Hematocrit (HCT) (%)	0.34	-0.10	0.77
		Mean Corpuscular Volume (MCV) (fl)	-0.32	-0.29	0.53
		Mean Corpuscular Hemoglobin (MCH) (pg)	0.03	0.54	-0.74
		Mean Corpuscular Hemoglobin Concentration (MCHC) (K/ul)	0.13	0.50	-0.77
	Thrombocyte Count	Platelets (PLT) (K/ul)	0.15	-0.25	0.67
RT-PCR	Inflammatory Markers	Tumor necrosis factor-α (TNF-α) ($2^{-\Delta Ct}$)	0.03	0.23	-0.19
		Interleukin-1β (IL-1β) ($2^{-\Delta Ct}$)	0.24	0.02	-0.17
		Interleukin-10 (IL-10) ($2^{-\Delta Ct}$)	0.00	0.07	-0.23
		Arginine (Arg) ($2^{-\Delta Ct}$)	-0.36	-0.58	0.07
Traumatic Brain Injury	Controlled Cortical Impact	CCI: Depth (mm)	-0.34	0.79	0.30
		CCI: Velocity (m/sec)	-0.33	0.79	0.31
	Brain Lesion	Gross Brain Lesion Area (%)	-0.42	0.75	0.09
		Total Brain Lesion Volume (%)	-0.25	0.82	0.35

PC Loading: -1 0 1

Supplementary Figure 4. Multivariate analysis of principal component solution. (A) PCA identified 7 PCs that were accepted using the eigenvalues greater than 1 as the Kaiser criterion. **(B)** The Cattell's Scree test suggested retaining PC1-3 for interpretation of PC content based on significant PC loading values. **(C)** PC over-determination criterion confirmed retaining PC1-3 with loading values above |0.6| in more than 5 outcome variables for the interpretation. PC loading is represented by heat (blue reflects negative and red reflects positive relationship between the individual variable and the loading value).
